# 1-Benzoyl-3-[3-cyano-8-methyl-4-(1-methyl-1*H*-pyrrol-2-yl)-5,6,7,8-tetra­hydro­quinolin-2-yl]thio­urea

**DOI:** 10.1107/S1600536811033046

**Published:** 2011-08-27

**Authors:** Abdullah M. Asiri, Hassan M. Faidallah, Abdulrahman O. Al-Youbi, Khalid A. Alamry, Seik Weng Ng

**Affiliations:** a Chemistry Department, Faculty of Science, King Abdulaziz University, PO Box 80203 Jeddah, Saudi Arabia; b Center of Excellence for Advanced Materials Research, King Abdulaziz University, PO Box 80203 Jeddah, Saudi Arabia; c Department of Chemistry, University of Malaya, 50603 Kuala Lumpur, Malaysia

## Abstract

In the *N*-substituted benzoyl­thio­urea, C_24_H_23_N_5_OS, the benzoyl­thio­urea unit is non-planar (r.m.s. deviation = 0.126 Å). The aliphatic part of the tetra­hydro­quinoline fused-ring system is disordered over two positions in a 0.592 (5):0.408 (5) ratio. The pyridine and pyrrole rings are twisted by 55.2 (1)° in order to avoid crowding of their respective substituents. Pairs of mol­ecules are linked by N—H⋯N hydrogen bonds, forming centrosymmetric dimers. Furthermore, an intra­molecular N—H⋯O hydrogen bond stabilizes the mol­ecular conformation.

## Related literature

For medicinal properties of cyano­pyridines, see: Cocco *et al.* (2005 ▶); El-Hawash *et al.* (2006 ▶).
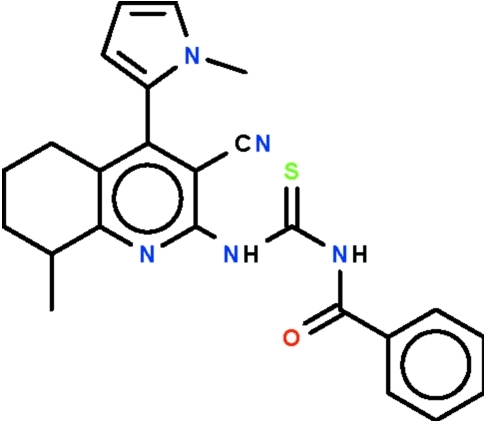


## Experimental

### 

#### Crystal data

C_24_H_23_N_5_OS
*M* *_r_* = 429.53
Triclinic, 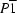

*a* = 9.7072 (4) Å
*b* = 10.4928 (5) Å
*c* = 11.8828 (5) Å
α = 82.245 (4)°
β = 84.263 (3)°
γ = 63.671 (4)°
*V* = 1073.76 (8) Å^3^
*Z* = 2
Cu *K*α radiation
μ = 1.55 mm^−1^
*T* = 100 K
0.30 × 0.20 × 0.20 mm

#### Data collection

Agilent SuperNova Dual diffractometer with an Atlas detector
Absorption correction: multi-scan (*CrysAlis PRO*; Agilent, 2010 ▶) *T*_min_ = 0.654, *T*_max_ = 0.747
7386 measured reflections
4218 independent reflections
3897 reflections with *I* > 2σ(*I*)
*R*_int_ = 0.020

#### Refinement

*R*[*F* ^2^ > 2σ(*F* ^2^)] = 0.052
*wR*(*F* ^2^) = 0.146
*S* = 1.03
4218 reflections
294 parameters
20 restraints
H-atom parameters constrained
Δρ_max_ = 1.25 e Å^−3^
Δρ_min_ = −0.46 e Å^−3^

### 

Data collection: *CrysAlis PRO* (Agilent, 2010 ▶); cell refinement: *CrysAlis PRO*; data reduction: *CrysAlis PRO*; program(s) used to solve structure: *SHELXS97* (Sheldrick, 2008 ▶); program(s) used to refine structure: *SHELXL97* (Sheldrick, 2008 ▶); molecular graphics: *X-SEED* (Barbour, 2001 ▶); software used to prepare material for publication: *publCIF* (Westrip, 2010 ▶).

## Supplementary Material

Crystal structure: contains datablock(s) global, I. DOI: 10.1107/S1600536811033046/bt5613sup1.cif

Structure factors: contains datablock(s) I. DOI: 10.1107/S1600536811033046/bt5613Isup2.hkl

Supplementary material file. DOI: 10.1107/S1600536811033046/bt5613Isup3.cml

Additional supplementary materials:  crystallographic information; 3D view; checkCIF report

## Figures and Tables

**Table 1:** Hydrogen-bond geometry (Å, °)

*D*—H⋯*A*	*D*—H	H⋯*A*	*D*⋯*A*	*D*—H⋯*A*
N4—H4⋯O1	0.88	1.90	2.594 (2)	135
N5—H5⋯N3^i^	0.88	2.22	3.058 (2)	158

Symmetry code: (i) 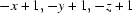.

**Fig. 1.**
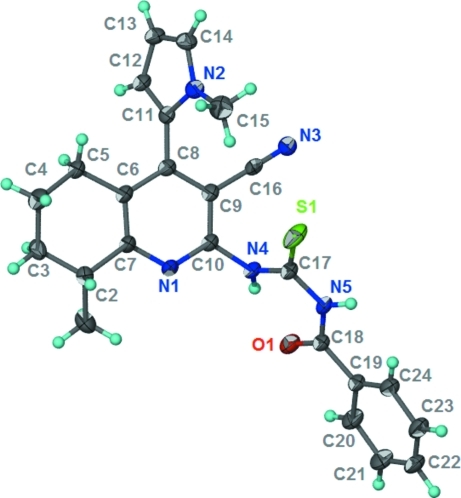
Anisotropic displacement ellipsoid plot (Barbour, 2001) of C24H23N5OS at the 70% probability level; hydrogen atoms are drawn as spheres of arbitrary radius. The disorder in the cyclohexene ring is not shown.

## supplementary crystallographic information

### Comment

There are several studies on cyanopyridine derivatives as these compounds exhibit useful anticancer and antiviral activities Cocco *et al.*, 2005; El-Hawash *et al.*, 2006). If these compounds possess a primary amine group, then they can be reacted with phenyl isothiocyanate to yield cyanopyridine-benzoythiourea derivatives, yet another class of medicinal compounds. Because of the ease phenyl isothiocyanate reacts with primary amines, we have in this study used 2-amino-3-cyano–8-methyl-4-(*N*-methylpyrrolyl)-5,6,7,8-tetrahydroquinoline to synthesize the correponding *N*-substituted benzoylthiourea (Scheme I).

In the *N*-substituted benzoylthiourea, C_24_H_23_N_5_OS, the benzoylthiourea portion is somewhat non-planar; the mean plane is aligned at 67.9 (1)° with respect the the mean-plane of the non-planar tetrahydroquinoline fused-ring. An intramolecular N–H···O hydrogen bond appears to prevent further twisting in the benzoylthiourea portion. The aliphatic portion of the tetrahydroquinoline fused-ring is disordered over two positions in a 0.592 (5): 0.408 ratio. The pyridine ring (which has a cyanide substituent) and the pyrrole ring (which has a methyl substitutent) are twisted by 55.2 (1) ° in order to avoid crowding of their respective substituents (Fig. 1). Two molecules are linked by an N–H···O hydrogen bonds to form a centrosymmetric dimer (Table 1).

### Experimental

2-Amino-3-cyano–8-methyl-4-(*N*-methylpyrrolyl)-5,6,7,8-tetrahydroquinoline (10 mmol), potassium carbonate (20 mmol) in dry acetone (25 ml) was stirred and then treated with phenyl isothiocyanate (12 mmol). The mixture was heated for 10 h; the acetone was removed under pressure and the solid mass dissolved in water. The solution was acidified with 2 N hydrochloric acid. The crude product was purified by recrystallization from ethanol.

### Refinement

Carbon- and nitrogen-bound H-atoms were placed in calculated positions [C–H 0.95 to 1.00, N–H 0.88 Å, *U*_iso_(H) 1.2–15*U*_eq_(C,*N*)] and were included in the refinement in the riding model approximation.

The three atoms of the cyclohexane ring that are not part of the fused system are disordered over two positions, as is the methyl substituent. For these four atoms, 1,2-related distances were restrained to 1.54±0.01 Å and 1,3-related ones to 2.51±0.01 Å. The displacement parameters of the primed atoms were set to those of the umprimed ones. The site occupation factor of the major component refined to 59.2 (5) %.

### Crystal data

**Table:**

C_24_H_23_N_5_OS	*Z* = 2
*M**_r_* = 429.53	*F*(000) = 452
Triclinic, *P*1	*D*_x_ = 1.329 Mg m^−^^3^
Hall symbol: -P 1	Cu *K*α radiation, λ = 1.54184 Å
*a* = 9.7072 (4) Å	Cell parameters from 4274 reflections
*b* = 10.4928 (5) Å	θ = 3.8–74.2°
*c* = 11.8828 (5) Å	µ = 1.55 mm^−^^1^
α = 82.245 (4)°	*T* = 100 K
β = 84.263 (3)°	Prism, brown–orange
γ = 63.671 (4)°	0.30 × 0.20 × 0.20 mm
*V* = 1073.76 (8) Å^3^	

### Data collection

**Table:**

Agilent SuperNova Dual diffractometer with an Atlas detector	4218 independent reflections
Radiation source: SuperNova (Cu) X-ray Source	3897 reflections with *I* > 2σ(*I*)
Mirror	*R*_int_ = 0.020
Detector resolution: 10.4041 pixels mm^-1^	θ_max_ = 74.3°, θ_min_ = 3.8°
ω scans	*h* = −11→8
Absorption correction: multi-scan (*CrysAlis PRO*; Agilent, 2010)	*k* = −12→13
*T*_min_ = 0.654, *T*_max_ = 0.747	*l* = −14→14
7386 measured reflections	

### Refinement

**Table:**

Refinement on *F*^2^	Primary atom site location: structure-invariant direct methods
Least-squares matrix: full	Secondary atom site location: difference Fourier map
*R*[*F*^2^ > 2σ(*F*^2^)] = 0.052	Hydrogen site location: inferred from neighbouring sites
*wR*(*F*^2^) = 0.146	H-atom parameters constrained
*S* = 1.03	*w* = 1/[σ^2^(*F*_o_^2^) + (0.0825*P*)^2^ + 0.7756*P*] where *P* = (*F*_o_^2^ + 2*F*_c_^2^)/3
4218 reflections	(Δ/σ)_max_ = 0.001
294 parameters	Δρ_max_ = 1.25 e Å^−^^3^
20 restraints	Δρ_min_ = −0.46 e Å^−^^3^

### Fractional atomic coordinates and isotropic or equivalent isotropic displacement parameters (Å^2^)

**Table:**

	*x*	*y*	*z*	*U*_iso_*/*U*_eq_	Occ. (<1)
S1	0.25749 (6)	0.66030 (6)	0.41290 (5)	0.03749 (18)	
O1	0.63636 (17)	0.62389 (15)	0.14578 (12)	0.0305 (3)	
N1	0.2933 (2)	1.01329 (17)	0.24918 (14)	0.0344 (4)	
N2	0.16227 (18)	1.03258 (17)	0.67653 (13)	0.0251 (3)	
N3	0.49398 (19)	0.73393 (17)	0.59776 (13)	0.0270 (4)	
N4	0.43635 (19)	0.77111 (16)	0.29593 (13)	0.0240 (3)	
H4	0.5199	0.7558	0.2524	0.029*	
N5	0.48814 (18)	0.54112 (16)	0.26536 (13)	0.0235 (3)	
H5	0.4773	0.4630	0.2898	0.028*	
C1	0.2196 (5)	1.2331 (4)	0.0699 (3)	0.0388 (8)	0.592 (5)
H1A	0.1607	1.3010	0.0078	0.058*	0.592 (5)
H1B	0.2515	1.1353	0.0516	0.058*	0.592 (5)
H1C	0.3109	1.2470	0.0796	0.058*	0.592 (5)
C2	0.1198 (4)	1.2577 (3)	0.1793 (3)	0.0238 (7)	0.592 (5)
H2	0.0328	1.2349	0.1681	0.029*	0.592 (5)
C3	0.0504 (4)	1.4093 (4)	0.2080 (4)	0.0286 (11)	0.592 (5)
H3A	−0.0277	1.4696	0.1520	0.034*	0.592 (5)
H3B	0.1320	1.4426	0.1998	0.034*	0.592 (5)
C4	−0.0245 (11)	1.4323 (12)	0.3259 (5)	0.0466 (10)	0.592 (5)
H4A	−0.1133	1.4088	0.3331	0.056*	0.592 (5)
H4B	−0.0628	1.5339	0.3389	0.056*	0.592 (5)
C1'	0.1144 (7)	1.2261 (6)	0.0836 (4)	0.0388 (8)	0.408
H1'A	0.0981	1.2943	0.0155	0.058*	0.408 (5)
H1'B	0.0150	1.2317	0.1156	0.058*	0.408 (5)
H1'C	0.1799	1.1291	0.0633	0.058*	0.408 (5)
C2'	0.1916 (6)	1.2618 (5)	0.1706 (4)	0.0238 (7)	0.408
H2'	0.2952	1.2502	0.1384	0.029*	0.408 (5)
C3'	0.1035 (7)	1.4112 (6)	0.2064 (5)	0.0286 (11)	0.408
H3'1	0.1780	1.4469	0.2214	0.034*	0.408 (5)
H3'2	0.0414	1.4746	0.1426	0.034*	0.408 (5)
C4'	−0.0017 (15)	1.4211 (19)	0.3106 (6)	0.0466 (10)	0.408
H4'1	−0.0741	1.3818	0.2978	0.056*	0.408 (5)
H4'2	−0.0628	1.5225	0.3244	0.056*	0.408 (5)
C5	0.0917 (2)	1.3376 (2)	0.41467 (16)	0.0295 (4)	
H5A	0.1619	1.3809	0.4228	0.035*	0.592 (5)
H5B	0.0354	1.3367	0.4888	0.035*	0.592 (5)
H5C	0.1599	1.3801	0.4302	0.035*	0.408 (5)
H5D	0.0233	1.3403	0.4829	0.035*	0.408 (5)
C6	0.1874 (2)	1.18481 (19)	0.38818 (16)	0.0255 (4)	
C7	0.2144 (3)	1.1494 (2)	0.27625 (16)	0.0357 (5)	
C8	0.2487 (2)	1.07401 (19)	0.47547 (15)	0.0217 (4)	
C9	0.3370 (2)	0.93424 (19)	0.44573 (15)	0.0223 (4)	
C10	0.3513 (2)	0.90956 (19)	0.33176 (16)	0.0249 (4)	
C11	0.2239 (2)	1.10237 (18)	0.59522 (15)	0.0220 (4)	
C12	0.2572 (2)	1.1937 (2)	0.64847 (16)	0.0253 (4)	
H12	0.3007	1.2552	0.6133	0.030*	
C13	0.2148 (2)	1.1790 (2)	0.76492 (16)	0.0298 (4)	
H13	0.2259	1.2276	0.8228	0.036*	
C14	0.1548 (2)	1.0819 (2)	0.77873 (16)	0.0291 (4)	
H14	0.1143	1.0532	0.8484	0.035*	
C15	0.0892 (2)	0.9433 (2)	0.65744 (19)	0.0334 (5)	
H15A	0.1105	0.8670	0.7200	0.050*	
H15B	0.1301	0.9011	0.5856	0.050*	
H15C	−0.0221	1.0016	0.6538	0.050*	
C16	0.4215 (2)	0.82116 (19)	0.53048 (15)	0.0225 (4)	
C17	0.3993 (2)	0.66125 (19)	0.32327 (16)	0.0244 (4)	
C18	0.5910 (2)	0.53169 (19)	0.17416 (15)	0.0233 (4)	
C19	0.6415 (2)	0.40659 (19)	0.10662 (15)	0.0244 (4)	
C20	0.7465 (3)	0.4002 (2)	0.01627 (18)	0.0352 (5)	
H20	0.7870	0.4686	0.0045	0.042*	
C21	0.7924 (3)	0.2941 (2)	−0.05700 (19)	0.0416 (6)	
H21	0.8637	0.2905	−0.1189	0.050*	
C22	0.7342 (3)	0.1942 (2)	−0.03949 (18)	0.0371 (5)	
H22	0.7655	0.1217	−0.0893	0.045*	
C23	0.6301 (3)	0.1997 (2)	0.05058 (18)	0.0334 (5)	
H23	0.5905	0.1306	0.0625	0.040*	
C24	0.5832 (2)	0.3061 (2)	0.12401 (16)	0.0281 (4)	
H24	0.5116	0.3097	0.1856	0.034*	

### Atomic displacement parameters (Å^2^)

**Table:**

	*U*^11^	*U*^22^	*U*^33^	*U*^12^	*U*^13^	*U*^23^
S1	0.0378 (3)	0.0290 (3)	0.0459 (3)	−0.0144 (2)	0.0180 (2)	−0.0197 (2)
O1	0.0407 (8)	0.0228 (7)	0.0261 (7)	−0.0125 (6)	0.0078 (6)	−0.0084 (5)
N1	0.0571 (11)	0.0163 (8)	0.0187 (8)	−0.0045 (8)	−0.0059 (7)	−0.0047 (6)
N2	0.0255 (8)	0.0218 (8)	0.0216 (8)	−0.0050 (6)	0.0024 (6)	−0.0033 (6)
N3	0.0295 (8)	0.0211 (8)	0.0220 (8)	−0.0038 (7)	0.0016 (6)	−0.0031 (6)
N4	0.0301 (8)	0.0163 (7)	0.0192 (7)	−0.0036 (6)	0.0019 (6)	−0.0063 (6)
N5	0.0296 (8)	0.0146 (7)	0.0213 (8)	−0.0047 (6)	0.0022 (6)	−0.0057 (6)
C1	0.055 (2)	0.0305 (15)	0.0249 (14)	−0.0156 (15)	−0.0034 (14)	0.0055 (11)
C2	0.028 (2)	0.0185 (11)	0.0216 (12)	−0.0078 (15)	−0.0025 (14)	−0.0007 (9)
C3	0.027 (3)	0.0208 (11)	0.0229 (11)	0.0015 (16)	0.0031 (17)	0.0022 (8)
C4	0.057 (3)	0.026 (2)	0.0306 (18)	0.0067 (18)	−0.0013 (17)	−0.0077 (17)
C1'	0.055 (2)	0.0305 (15)	0.0249 (14)	−0.0156 (15)	−0.0034 (14)	0.0055 (11)
C2'	0.028 (2)	0.0185 (11)	0.0216 (12)	−0.0078 (15)	−0.0025 (14)	−0.0007 (9)
C3'	0.027 (3)	0.0208 (11)	0.0229 (11)	0.0015 (16)	0.0031 (17)	0.0022 (8)
C4'	0.057 (3)	0.026 (2)	0.0306 (18)	0.0067 (18)	−0.0013 (17)	−0.0077 (17)
C5	0.0386 (11)	0.0161 (9)	0.0231 (9)	−0.0013 (8)	−0.0022 (8)	−0.0050 (7)
C6	0.0326 (10)	0.0163 (9)	0.0215 (9)	−0.0041 (7)	−0.0028 (7)	−0.0050 (7)
C7	0.0585 (14)	0.0157 (9)	0.0199 (9)	−0.0030 (9)	−0.0064 (9)	−0.0036 (7)
C8	0.0233 (8)	0.0188 (9)	0.0193 (9)	−0.0050 (7)	−0.0004 (7)	−0.0049 (7)
C9	0.0247 (9)	0.0173 (9)	0.0196 (9)	−0.0042 (7)	−0.0002 (7)	−0.0032 (7)
C10	0.0317 (10)	0.0166 (9)	0.0212 (9)	−0.0047 (7)	−0.0009 (7)	−0.0056 (7)
C11	0.0225 (8)	0.0165 (8)	0.0189 (8)	−0.0014 (7)	0.0013 (6)	−0.0030 (6)
C12	0.0300 (9)	0.0189 (9)	0.0208 (9)	−0.0045 (7)	0.0022 (7)	−0.0068 (7)
C13	0.0363 (10)	0.0239 (10)	0.0196 (9)	−0.0034 (8)	0.0000 (7)	−0.0073 (7)
C14	0.0314 (10)	0.0279 (10)	0.0172 (9)	−0.0038 (8)	0.0042 (7)	−0.0036 (7)
C15	0.0312 (10)	0.0335 (11)	0.0349 (11)	−0.0145 (9)	0.0041 (8)	−0.0043 (9)
C16	0.0246 (9)	0.0182 (9)	0.0193 (8)	−0.0043 (7)	0.0048 (7)	−0.0070 (7)
C17	0.0282 (9)	0.0169 (9)	0.0214 (9)	−0.0029 (7)	−0.0008 (7)	−0.0059 (7)
C18	0.0274 (9)	0.0168 (8)	0.0191 (8)	−0.0030 (7)	−0.0018 (7)	−0.0043 (7)
C19	0.0284 (9)	0.0167 (9)	0.0199 (9)	−0.0011 (7)	−0.0031 (7)	−0.0049 (7)
C20	0.0436 (12)	0.0252 (10)	0.0298 (10)	−0.0087 (9)	0.0092 (9)	−0.0109 (8)
C21	0.0521 (14)	0.0286 (11)	0.0304 (11)	−0.0050 (10)	0.0106 (10)	−0.0129 (9)
C22	0.0472 (12)	0.0226 (10)	0.0279 (10)	0.0007 (9)	−0.0066 (9)	−0.0125 (8)
C23	0.0417 (11)	0.0218 (10)	0.0322 (11)	−0.0062 (9)	−0.0093 (9)	−0.0096 (8)
C24	0.0344 (10)	0.0222 (9)	0.0230 (9)	−0.0064 (8)	−0.0041 (8)	−0.0065 (7)

### Geometric parameters (Å, °)

**Table:**

S1—C17	1.657 (2)	C3'—H3'2	0.9900
O1—C18	1.226 (2)	C4'—C5	1.532 (9)
N1—C10	1.320 (2)	C4'—H4'1	0.9900
N1—C7	1.354 (2)	C4'—H4'2	0.9900
N2—C14	1.366 (3)	C5—C6	1.513 (2)
N2—C11	1.382 (2)	C5—H5A	0.9900
N2—C15	1.452 (3)	C5—H5B	0.9900
N3—C16	1.147 (2)	C5—H5C	0.9900
N4—C17	1.344 (3)	C5—H5D	0.9900
N4—C10	1.416 (2)	C6—C7	1.398 (3)
N4—H4	0.8800	C6—C8	1.402 (3)
N5—C18	1.380 (2)	C8—C9	1.405 (2)
N5—C17	1.399 (2)	C8—C11	1.468 (2)
N5—H5	0.8800	C9—C10	1.395 (3)
C1—C2	1.521 (4)	C9—C16	1.440 (2)
C1—H1A	0.9800	C11—C12	1.379 (3)
C1—H1B	0.9800	C12—C13	1.412 (3)
C1—H1C	0.9800	C12—H12	0.9500
C2—C3	1.500 (5)	C13—C14	1.366 (3)
C2—C7	1.547 (3)	C13—H13	0.9500
C2—H2	1.0000	C14—H14	0.9500
C3—C4	1.511 (6)	C15—H15A	0.9800
C3—H3A	0.9900	C15—H15B	0.9800
C3—H3B	0.9900	C15—H15C	0.9800
C4—C5	1.527 (7)	C18—C19	1.497 (2)
C4—H4A	0.9900	C19—C24	1.386 (3)
C4—H4B	0.9900	C19—C20	1.391 (3)
C1'—C2'	1.510 (6)	C20—C21	1.393 (3)
C1'—H1'A	0.9800	C20—H20	0.9500
C1'—H1'B	0.9800	C21—C22	1.380 (4)
C1'—H1'C	0.9800	C21—H21	0.9500
C2'—C3'	1.513 (6)	C22—C23	1.385 (3)
C2'—C7	1.563 (5)	C22—H22	0.9500
C2'—H2'	1.0000	C23—C24	1.396 (3)
C3'—C4'	1.508 (9)	C23—H23	0.9500
C3'—H3'1	0.9900	C24—H24	0.9500
			
C10—N1—C7	118.70 (16)	C6—C5—H5C	109.8
C14—N2—C11	108.27 (16)	C4—C5—H5C	111.7
C14—N2—C15	123.49 (17)	C4'—C5—H5C	109.7
C11—N2—C15	127.26 (17)	C6—C5—H5D	110.1
C17—N4—C10	125.08 (16)	C4—C5—H5D	101.5
C17—N4—H4	117.5	C4'—C5—H5D	111.1
C10—N4—H4	117.5	H5C—C5—H5D	108.3
C18—N5—C17	127.16 (16)	C7—C6—C8	117.99 (17)
C18—N5—H5	116.4	C7—C6—C5	121.05 (17)
C17—N5—H5	116.4	C8—C6—C5	120.94 (16)
C2—C1—H1A	109.5	N1—C7—C6	122.96 (17)
C2—C1—H1B	109.5	N1—C7—C2	114.67 (19)
H1A—C1—H1B	109.5	C6—C7—C2	120.51 (19)
C2—C1—H1C	109.5	N1—C7—C2'	112.4 (2)
H1A—C1—H1C	109.5	C6—C7—C2'	123.2 (2)
H1B—C1—H1C	109.5	C6—C8—C9	118.42 (16)
C3—C2—C1	113.7 (3)	C6—C8—C11	121.25 (16)
C3—C2—C7	112.8 (3)	C9—C8—C11	120.33 (16)
C1—C2—C7	108.8 (3)	C10—C9—C8	118.92 (16)
C3—C2—H2	107.1	C10—C9—C16	120.70 (16)
C1—C2—H2	107.1	C8—C9—C16	120.18 (16)
C7—C2—H2	107.1	N1—C10—C9	122.84 (17)
C2—C3—C4	115.0 (5)	N1—C10—N4	115.13 (16)
C2—C3—H3A	108.5	C9—C10—N4	121.96 (16)
C4—C3—H3A	108.5	C12—C11—N2	107.94 (16)
C2—C3—H3B	108.5	C12—C11—C8	129.53 (17)
C4—C3—H3B	108.5	N2—C11—C8	122.51 (17)
H3A—C3—H3B	107.5	C11—C12—C13	107.41 (18)
C3—C4—C5	110.0 (5)	C11—C12—H12	126.3
C3—C4—H4A	109.7	C13—C12—H12	126.3
C5—C4—H4A	109.7	C14—C13—C12	107.14 (17)
C3—C4—H4B	109.7	C14—C13—H13	126.4
C5—C4—H4B	109.7	C12—C13—H13	126.4
H4A—C4—H4B	108.2	N2—C14—C13	109.21 (17)
C2'—C1'—H1'A	109.5	N2—C14—H14	125.4
C2'—C1'—H1'B	109.5	C13—C14—H14	125.4
H1'A—C1'—H1'B	109.5	N2—C15—H15A	109.5
C2'—C1'—H1'C	109.5	N2—C15—H15B	109.5
H1'A—C1'—H1'C	109.5	H15A—C15—H15B	109.5
H1'B—C1'—H1'C	109.5	N2—C15—H15C	109.5
C1'—C2'—C3'	114.5 (4)	H15A—C15—H15C	109.5
C1'—C2'—C7	106.8 (4)	H15B—C15—H15C	109.5
C3'—C2'—C7	109.8 (4)	N3—C16—C9	176.5 (2)
C1'—C2'—H2'	108.5	N4—C17—N5	114.65 (16)
C3'—C2'—H2'	108.5	N4—C17—S1	125.98 (14)
C7—C2'—H2'	108.5	N5—C17—S1	119.31 (14)
C4'—C3'—C2'	114.1 (8)	O1—C18—N5	122.12 (16)
C4'—C3'—H3'1	108.7	O1—C18—C19	120.29 (17)
C2'—C3'—H3'1	108.7	N5—C18—C19	117.55 (16)
C4'—C3'—H3'2	108.7	C24—C19—C20	119.68 (18)
C2'—C3'—H3'2	108.7	C24—C19—C18	124.42 (17)
H3'1—C3'—H3'2	107.6	C20—C19—C18	115.74 (18)
C3'—C4'—C5	110.5 (8)	C19—C20—C21	120.4 (2)
C3'—C4'—H4'1	109.6	C19—C20—H20	119.8
C5—C4'—H4'1	109.6	C21—C20—H20	119.8
C3'—C4'—H4'2	109.6	C22—C21—C20	119.8 (2)
C5—C4'—H4'2	109.6	C22—C21—H21	120.1
H4'1—C4'—H4'2	108.1	C20—C21—H21	120.1
C6—C5—C4	114.9 (4)	C21—C22—C23	120.07 (19)
C6—C5—C4'	107.8 (6)	C21—C22—H22	120.0
C4—C5—C4'	10.0 (5)	C23—C22—H22	120.0
C6—C5—H5A	108.5	C22—C23—C24	120.4 (2)
C4—C5—H5A	108.5	C22—C23—H23	119.8
C4'—C5—H5A	105.9	C24—C23—H23	119.8
C6—C5—H5B	108.5	C19—C24—C23	119.7 (2)
C4—C5—H5B	108.5	C19—C24—H24	120.2
C4'—C5—H5B	118.2	C23—C24—H24	120.2
H5A—C5—H5B	107.5		
			
C1—C2—C3—C4	−169.1 (6)	C7—N1—C10—C9	−1.1 (3)
C7—C2—C3—C4	−44.7 (7)	C7—N1—C10—N4	−178.1 (2)
C2—C3—C4—C5	56.7 (12)	C8—C9—C10—N1	4.3 (3)
C1'—C2'—C3'—C4'	92.8 (9)	C16—C9—C10—N1	−170.5 (2)
C7—C2'—C3'—C4'	−27.3 (9)	C8—C9—C10—N4	−178.92 (18)
C2'—C3'—C4'—C5	64.7 (16)	C16—C9—C10—N4	6.2 (3)
C3—C4—C5—C6	−45.1 (12)	C17—N4—C10—N1	−117.5 (2)
C3—C4—C5—C4'	0(8)	C17—N4—C10—C9	65.6 (3)
C3'—C4'—C5—C6	−57.1 (15)	C14—N2—C11—C12	1.0 (2)
C3'—C4'—C5—C4	166 (10)	C15—N2—C11—C12	169.90 (18)
C4—C5—C6—C7	25.2 (6)	C14—N2—C11—C8	179.69 (16)
C4'—C5—C6—C7	17.8 (8)	C15—N2—C11—C8	−11.5 (3)
C4—C5—C6—C8	−153.3 (6)	C6—C8—C11—C12	−56.1 (3)
C4'—C5—C6—C8	−160.7 (8)	C9—C8—C11—C12	123.0 (2)
C10—N1—C7—C6	−2.0 (4)	C6—C8—C11—N2	125.6 (2)
C10—N1—C7—C2	−166.5 (2)	C9—C8—C11—N2	−55.3 (3)
C10—N1—C7—C2'	164.6 (3)	N2—C11—C12—C13	0.0 (2)
C8—C6—C7—N1	1.6 (4)	C8—C11—C12—C13	−178.49 (18)
C5—C6—C7—N1	−176.9 (2)	C11—C12—C13—C14	−1.1 (2)
C8—C6—C7—C2	165.3 (2)	C11—N2—C14—C13	−1.8 (2)
C5—C6—C7—C2	−13.2 (4)	C15—N2—C14—C13	−171.12 (18)
C8—C6—C7—C2'	−163.5 (3)	C12—C13—C14—N2	1.7 (2)
C5—C6—C7—C2'	17.9 (4)	C10—C9—C16—N3	100 (3)
C3—C2—C7—N1	−172.9 (3)	C8—C9—C16—N3	−75 (3)
C1—C2—C7—N1	−45.7 (4)	C10—N4—C17—N5	171.52 (17)
C3—C2—C7—C6	22.2 (4)	C10—N4—C17—S1	−5.9 (3)
C1—C2—C7—C6	149.3 (3)	C18—N5—C17—N4	−10.1 (3)
C3—C2—C7—C2'	−81.8 (5)	C18—N5—C17—S1	167.55 (15)
C1—C2—C7—C2'	45.4 (5)	C17—N5—C18—O1	13.3 (3)
C1'—C2'—C7—N1	55.5 (4)	C17—N5—C18—C19	−164.14 (17)
C3'—C2'—C7—N1	−179.8 (4)	O1—C18—C19—C24	−172.29 (18)
C1'—C2'—C7—C6	−138.0 (4)	N5—C18—C19—C24	5.2 (3)
C3'—C2'—C7—C6	−13.2 (5)	O1—C18—C19—C20	3.2 (3)
C1'—C2'—C7—C2	−45.2 (5)	N5—C18—C19—C20	−179.29 (17)
C3'—C2'—C7—C2	79.6 (6)	C24—C19—C20—C21	0.4 (3)
C7—C6—C8—C9	1.6 (3)	C18—C19—C20—C21	−175.3 (2)
C5—C6—C8—C9	−179.84 (18)	C19—C20—C21—C22	−0.4 (4)
C7—C6—C8—C11	−179.3 (2)	C20—C21—C22—C23	0.1 (3)
C5—C6—C8—C11	−0.7 (3)	C21—C22—C23—C24	0.2 (3)
C6—C8—C9—C10	−4.4 (3)	C20—C19—C24—C23	−0.1 (3)
C11—C8—C9—C10	176.45 (18)	C18—C19—C24—C23	175.20 (18)
C6—C8—C9—C16	170.46 (18)	C22—C23—C24—C19	−0.2 (3)
C11—C8—C9—C16	−8.7 (3)		

### Hydrogen-bond geometry (Å, °)

**Table:**

*D*—H···*A*	*D*—H	H···*A*	*D*···*A*	*D*—H···*A*
N4—H4···O1	0.88	1.90	2.594 (2)	135
N5—H5···N3^i^	0.88	2.22	3.058 (2)	158

Symmetry codes: (i) −*x*+1, −*y*+1, −*z*+1.
